# Balancing workload, motivation and job satisfaction in Rwanda: assessing the effect of adding family planning service provision to community health worker duties

**DOI:** 10.1186/s12978-015-0110-z

**Published:** 2016-01-06

**Authors:** Dawn Chin-Quee, Cathy Mugeni, Denis Nkunda, Marie Rose Uwizeye, Laurie L. Stockton, Jennifer Wesson

**Affiliations:** 1 Health Services Research, FHI 360, 359 Blackwell St., Suite 200, Durham, NC 27701 USA; 2 Division of Community Health, Ministry of Health, Kigali, Rwanda; 3 Research in Social, Behavior and Health, Ltd., St. Paul Centre, Kigali, Rwanda; 4 School of Media and Journalism, University of North Carolina, Chapel Hill, NC 27599 USA; 5 IntraHealth International, 6340 Quadrangle Drive, Suite 200, Chapel Hill, 27517 NC USA

**Keywords:** Family planning, Community health workers, Motivation, Workload

## Abstract

**Background:**

Task shifting from higher cadre providers to CHWs has been widely adopted to address healthcare provider shortages, but the addition of any service can potentially add to an already considerable workload for CHWs. Objective measures of workload alone, such as work-related time and travel may not reflect howCHWs actually perceive and react to their circumstances. This study combined perception and objectivemeasures of workload to examine their effect on quality of services, worker performance, and job and clientsatisfaction.

**Methods:**

Three hundred eighty-three CHWs from control and intervention districts, where the intervention group was trained to provide contraceptive resupply, completed diaries of work-related activities for one month. Interviews were also conducted with a subset of CHWs and their clients.

**Results:**

CHW diaries did not reveal significant differences between intervention and control groups in time spent on service provision or travel. Over 90 % of CHWs reported workload manageability, job satisfaction, and motivation to perform their jobs. Clients were highly satisfied with CHW services and most stated preference for future services from CHWs.

**Conclusion:**

The study demonstrated that adding resupply of hormonal contraceptives to CHWs’ tasks would not place undue burden on them. Accordingly, the initiative was scaled up in all 30 districts in the country.

## Background

Task-shifting responsibilities to community health workers (CHWs) is seen as the solution to many human resource challenges in the health care system [1–3]. To compensate for low numbers of formally-trained providers, services such as immunizations; sanitation and hygiene; antenatal; maternal and child health care; infectious diseases and HIV/AIDS are now routinely provided by paid or volunteer CHWs. Family planning (FP) service provision by CHWs is widely recognized as an effective way to expand access to contraception at the community level [4]. In Uganda, Madagascar, Kenya, Senegal, Ethiopia, and Zambia, CHWs supported by non-governmental organizations and/or by the public sector initiate and re-supply pills, and sometimes injectable contraceptives [5–8].

The addition of any service can potentially add to an already considerable workload for CHWs, possibly resulting in decreased service quality and in discontent [9]. Health Surveillance Assistants (HSAs), the lowest cadre of paid Ministry of Health (MOH) health care providers in Malawi, provide most of the country’s primary health care services. In interviews conducted one year after initiation of HSA provision of the injectable contraception, Depo Provera® (DMPA), 40 % of HSAs reported that providing DMPA, in addition to their other duties created challenges in providing these services to far away clients [10]. Volunteer ChildFund CBD agents in a pilot study in Zambia also reported increases in their workload after they were trained to provide DMPA injections, but they did not report feeling overburdened [11].

The experiences in Malawi and Zambia illustrate that worker perception provides context to the issue of workload and that objective measures of workload alone, such as increases in work-related time and travel may not reflect how CHWs actually perceive and react to their circumstances. In this cadre of worker--whether volunteer as in Zambia or paid as in Malawi--perception and objective measures of workload have rarely been combined to examine their effect on quality of services, worker performance, and job and client satisfaction. However, with the emerging trend of shifting health care tasks to community-based workers, understanding worker perceptions in light of objective measures of workload will become increasingly important and may provide insight into issues of workload pressure, dropout rates, and incentives [12, 13].

The Rwandan MoH manages a nationwide network of volunteer CHWs—three per village--who provide a variety of services to their community. One CHW is in charge of maternal and child health and the other two comprise a binome. Binomes--a male and female pair of CHWs in each village--are tasked with providing community-based integrated management of child illness; growth monitoring of infants and children; family planning counseling, referral and condom provision; directly observed treatment of tuberculosis; rapid tests for malaria diagnosis; nutrition counseling; counseling on sanitation and home hygiene; as well as HIV/AIDS prevention and support for people living with HIV/AIDS.

Rwandan CHWs are attached to health centers which provide supportive supervision, training, commodities, and supplies. CHWs are also organized in cooperatives to ensure accountability and income generation. The Government makes payments to cooperatives upon receipt of proof of performance in a system called community performance-based financing. The cooperatives can then invest the earnings in income-generating projects such as animal husbandry, farming, or basket making. This government-sponsored system of incentives is designed to provide motivation to become and remain a CHW.

The MOH initiated a pilot program of community-based provision (CBP) of FP in March 2010 in three districts: Kicukiro, Rusizi and Gatsibo. Re-supply of oral contraceptive pills and DMPA were added to binomes’ existing offerings. Trained binome pairs in each village of the three districts commenced offering the enhanced service of pill and DMPA resupply in December 2010. Before undertaking a phased scale up in the remaining 27 districts in the country, the MoH wanted to assess if adding pill and DMPA re-supply would unduly burden binomes and affect quality of services. This was especially important if limited resources for scale up would allow only one person in the binome pair to be trained. With technical assistance from FHI 360, the MOH conducted a study to compare districts with enhanced FP service provision to districts that had not yet implemented re-supply of pills and DMPA. The specific objectives of the study were to compare intervention and control districts vis-à-vis CHWs’ work-related activities, their perceptions of workload manageability, reports of job satisfaction, motivation, and service quality, as well as their clients’ reports of satisfaction and quality of care.

## Methods

The MOH selected two of the three districts in which CHWs had been trained in CBP of FP (intervention: Kicukiro and Rusizi) to be compared with two whose CHWs had not been trained (control: Kayonza and Gasabo). Rusizi and Kayonza are rural districts, while Kicukiro and Gasabo are urban, ensuring that both types of districts were represented in control and intervention groups. In April 2012 there were 7 and 8 health centers in Rusizi and Kicukiro, respectively. One health center in Kicukiro, affiliated with the Catholic Church, was not included in the sample because it did not provide FP services. In the remaining 14 health centers, there were 910 CHWs in Rusizi and 582 in Kicukiro for a total of 1492 intervention binomes. There were 12 health centers in Gasabo and 13 in Kayonza with 874 and 1553 CHWs, respectively, for a total of 2427 control binomes.

**Fig. 1:**
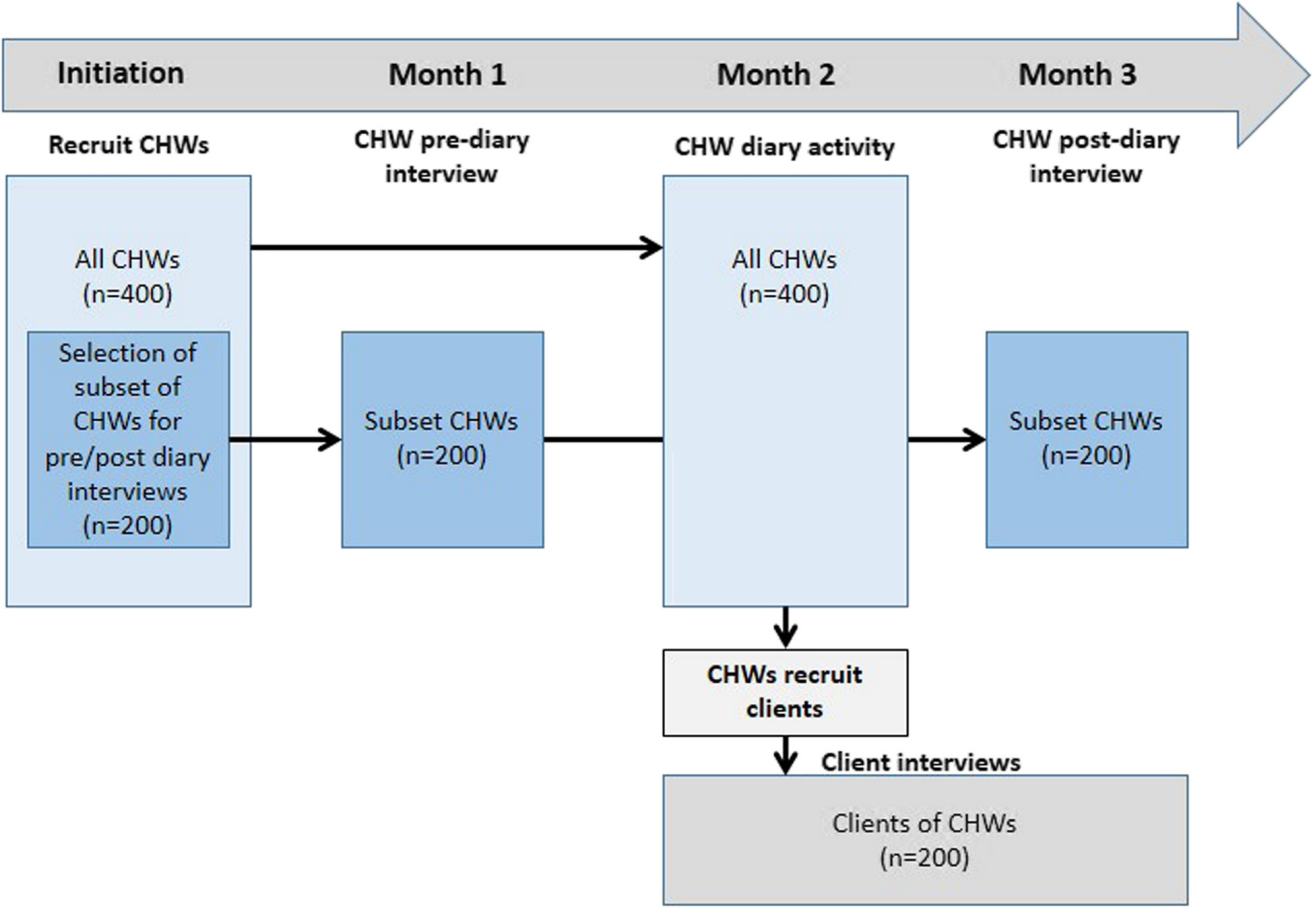
Timeline of study activities

We employed longitudinal and cross-sectional data collection methods from May to mid-July 2012 (Fig. 1). To capture information on CHW workload, 400 CHWs--200 from each study group--were randomly selected to record their daily activities for one month. Within each study group, the 200 were distributed proportionally to the total number of binomes in the final sampling frame in each of the two districts. In the intervention group, 61 % of the CHWs were selected from Rusizi; 39 % from Kicukiro. In the control group, 36 % of CHWs were selected from Gasabo and 64 % from Kayonza. Of the 400 CHWs, 100 were randomly selected to also participate in structured interviews. In both cases, CHWs were oversampled to account for refusals and attrition: 450 to record work-related activities and 120 for interviews.

To record their daily activities, CHWs were provided with a diary and training. CHWs were also asked to recruit clients who recently received services for interviews with study staff and record the contact information. In addition to a one-time recording of CHW’s demographic information (age, marital status, educational attainment, number of children, and length of CHW service), diaries solicited daily information on the number of clients/households visited or received in their homes, time spent with client/household, and services rendered to clients. Study staff collected diary information from the field after two weeks and at the end of the month to confirm that data were being recorded correctly and to extract contact information of clients who agreed to be interviewed.

Information on CHW perceptions of workload, job satisfaction, and sources of motivation were obtained from a semi-structured interview instrument administered to the subset of 100 CHWs. This proportion was selected due to logistic and resource constraints. The interviews were conducted with the CHWs before the diary-keeping exercise.

A random sample of 200 clients (i.e., approximately 50 in each district) were selected by FHI 360 staff in Kigali, applying a random number generator to the list of contacts provided by the CHWs in their diaries. Clients receiving any recent service were eligible and they were asked about the type of service(s) received, quality of the service(s), and overall satisfaction. One-half of the clients in each district were interviewed shortly after the first extraction of contact information from CHW diaries and the rest after the second extraction.

Diary data were entered in EpiData, version 3.1 (The EpiData Association, Odense Denmark, 2004) and structured interview data were collected using personal digital assistants. Both sets of data were converted to and analyzed with SPSS, version 17.0 (SPSS Inc. Chicago, 2008).

The study protocol and materials were approved by FHI 360’s Protection of Human Subjects Committee and the Rwanda National Ethics Committee.

## Results

Attrition over the course of data collection resulted in 188 CHWs in the intervention group and 195 in control districts who provided complete diary data. In control districts, there was a higher proportion of female CHWs, who had served a little longer as CHWs, were a bit younger and less likely to be in union than their counterparts in intervention districts. However, only the difference in mean age was significant (*p* < 0.05). CHWs in intervention and control groups had similar educational attainment and number of children (Table 1).

**Table 1:** Demographic characteristics of CHWs who completed diaries

Demographic characteristic	Control (*n* = 192)^a^	Intervention (*n* = 188)
Female (%)	64	58
Age (average in years)	39	41
Highest educational level completed (%)		
Primary	77	75
Secondary	4	8
Vocational	19	17
Married (%)	80	87
Average number of children	4.1	4.4
Mean number of years as a CHW	6.2	5.8

^a^ 3 CHWs in the control group who completed diaries did not provide information on one or more demographic characteristics

CHWs in both groups served about 40 clients per week, which amounted to approximately 20 h of work per week. About 13 % of clients from both control and intervention districts were FP clients. Table 2 shows the number of hours per week spent on work-related activities. Control district CHWs reported spending significantly more time on average on book-keeping or administrative tasks (not including study-related activities) than intervention CHWs (*p* < 0.05). However CHWs in the intervention group reported significantly more time spent on meetings, training, and supervision (*p* < 0.01), as well as community sensitization (*p* < 0.05). Intervention CHWs consistently reported spending more time per week with clients in large group counseling sessions on topics such as home hygiene, nutrition, sanitation and HIV/AIDS prevention than their control counterparts (data not shown). There were no significant differences between the two in terms of time spent providing individual services to clients or travel to and from clinic and home visits.

**Table 2:** Weekly time in hours spent on CHW work-related activities by study group

Activity	Control (*N* = 195)		Intervention (*N* = 188)		Test statistic
	Mean	Range	Mean	Range	p-value
Providing individual services to clients	6.1	0.5–21.2	6.5	0.5–25.0	0.320
Book-keeping/administrative tasks	2.2	0–24.5	1.7	0–7.8	0.012^a^
Meetings, training, and supervision	4.1	0–29.6	5.8	0–36.2	0.005^a^
Community sensitization	0.9	0–8.2	1.2	0–7.0	0.049^a^
Travel to/from clinic and home visits	5.6	0–20.4	5.6	0–16.3	0.848
Other work-related activities	1.7	0–18.7	2.7	0–39.7	0.059
Total time spent overall each month per CHW	20.5	2.0–67.8	19.4	2.1–50.8	0.054

^a^Indicates a significant difference between the control and intervention group’s time spent on that activity

Of the 120 CHWs sampled for interviews, 101 provided complete data: 50 from control and 51 from intervention districts. Demographic characteristics of this subset of CHWs were similar to the larger group of 383 CHWs and there were no significant differences between the intervention and control subgroups (data not shown).

**Fig. 2:**
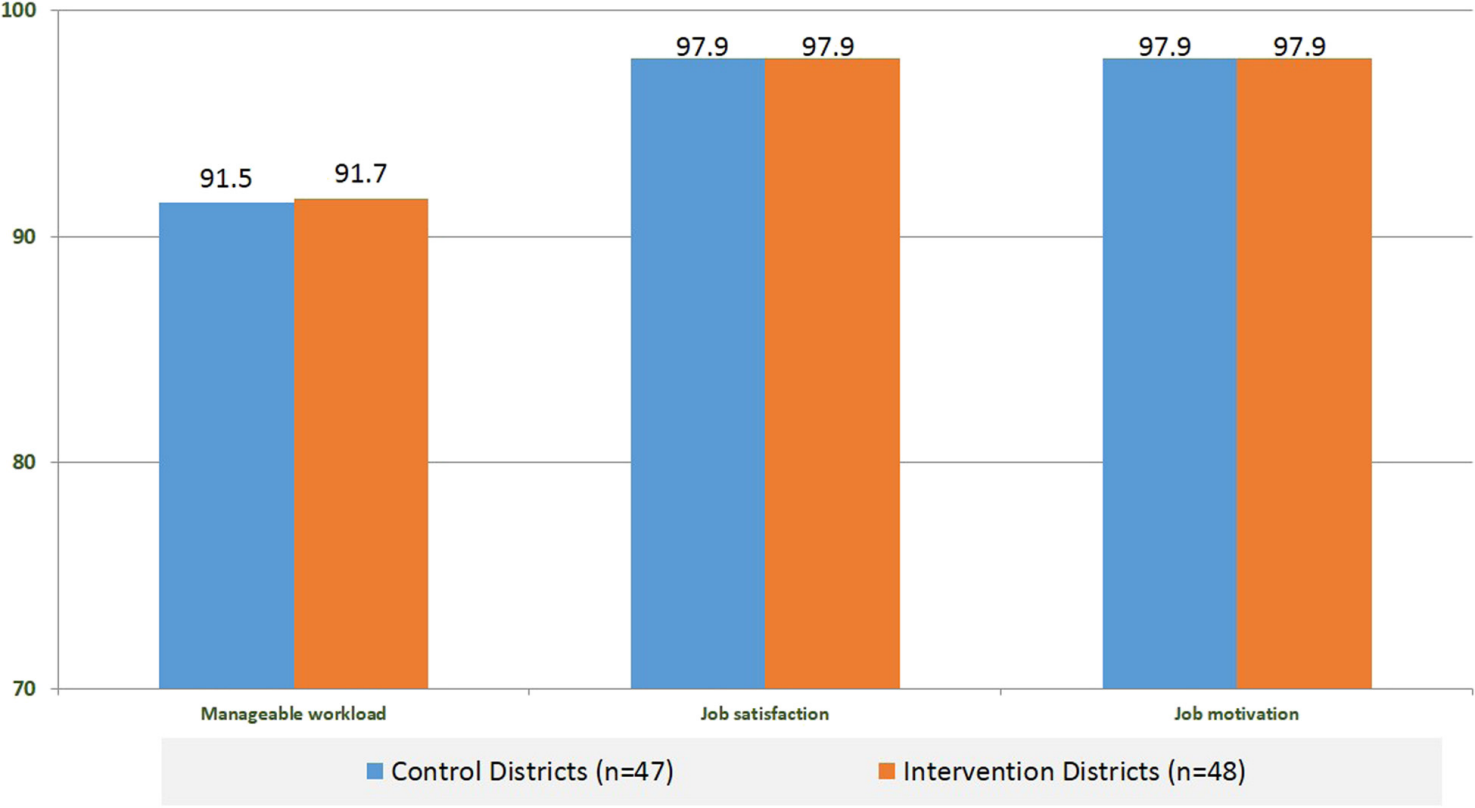
Percentage of CHWs reporting that their workload is manageable, they are satisfied with and motivated to perform their job

CHWs in both intervention and control groups reported that their workload was manageable and that job satisfaction and motivation were high (Fig. 2). The top three challenges for both groups were having too much responsibility (47 % intervention, 34 % control), not having enough time for family or self (33 % intervention, 40 % control), and lack of supplies/commodities (29 % intervention, 18 % control). There were no significant differences between groups. The top three sources of motivation were desire to help their community (81 % intervention, 77 % control), being able to gain new skills/knowledge (56 % intervention, 60 % control), and maintaining status in the community (58 % intervention, 47 % control). Again, there were no significant differences between the groups. Receiving incentives/material goods and earning income through their cooperatives were fairly low on the list of motivating factors for both groups.

In the intervention districts, 80 % reported an increase in client load since they started providing enhanced FP services, but none said that the additional tasks had a negative effect on their ability to provide services to all their clients. Nevertheless, only 39 % believed that FP needs could be met adequately by just one person in a binome pair.

The clients of CHWs were overwhelmingly female, in their 30s, and more than 70 % were in union (Table 3). With just over half of clients completing primary school, their educational attainment was generally lower than CHWs. Clients were also younger on average and had fewer children than CHWs.

**Table 3:** Demographic characteristics of CHW clients by study group

Characteristic	Control (*n* = 102)	Intervention (*n* = 98)
Female (%)	82	87
Age (average in years)	36	34
Highest level of education completed (%)		
Primary	52	54
Secondary	5	8
Vocational	3	6
Married (%)	73	79
Average number of children	3.3	3.7

**Fig. 3:**
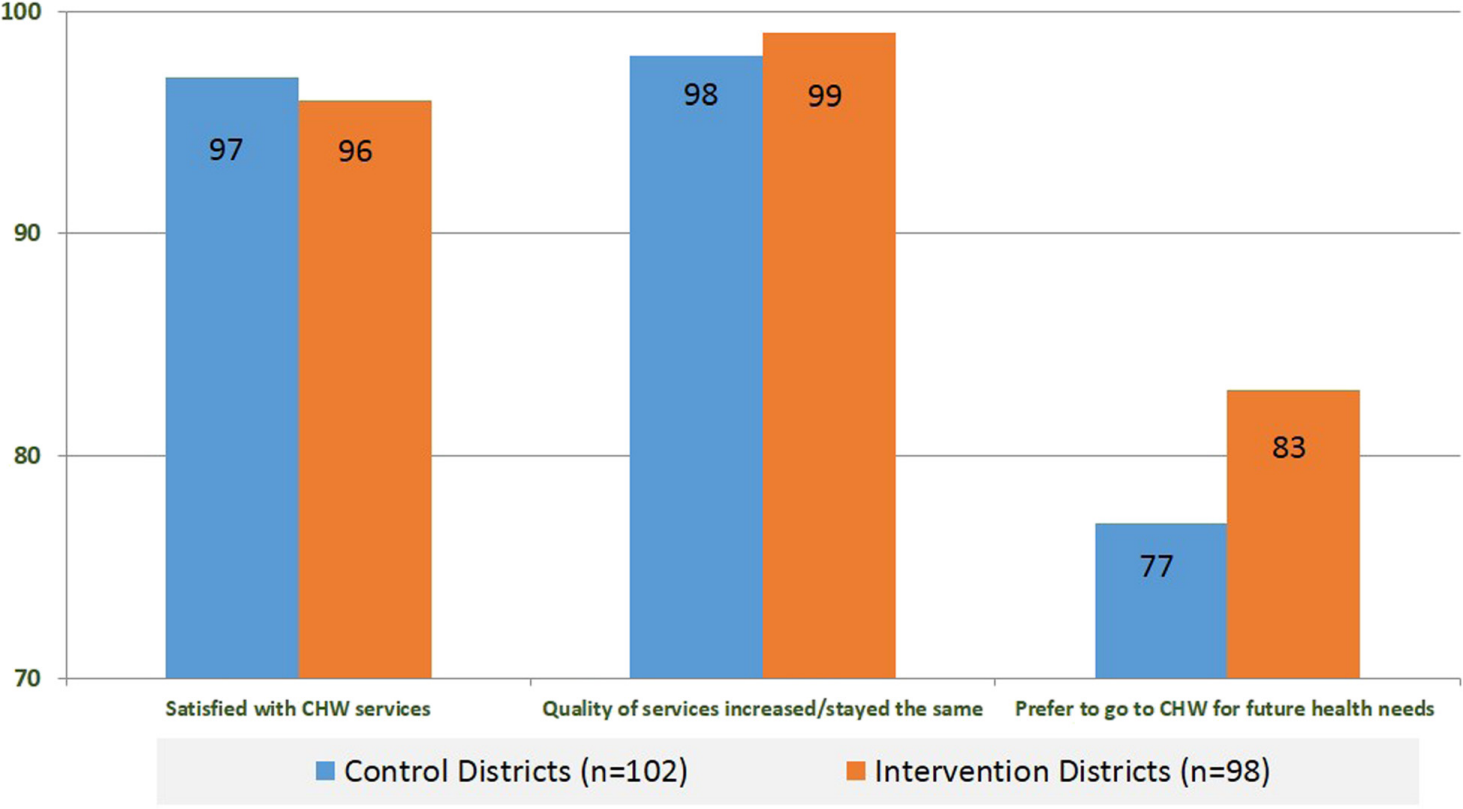
Percentage of clients reporting satisfaction with CHW services, increase or maintenance of service quality over time, and preference for CHW services for future health needs

CHW clients indicated that they were very satisfied with the service, regardless of CHW assignment. Clients received all the services offered by CHWs but mostly counseling in FP, nutrition and sanitation/home hygiene (data not shown). Almost all clients reported that the quality of CHW services increased or stayed the same over the past year and a smaller majority also reported that they would prefer to go to their CHW for future health needs (Fig. 3). Aspects of client-provider interaction were also overwhelmingly positive as more than 90 % of clients in both groups reported that CHWs devoted the time and attention needed, answered all their questions, addressed them in a friendly way, and could be trusted to protect their privacy (data not shown).

## Discussion and conclusions

CHWs who resupply pills and injectable contraception did not report a more unmanageable workload than their counterparts. They also reported similar levels of job satisfaction and motivation as CHWs who did not provide these enhanced services, suggesting that adding FP provision did not create an undue burden. These results were encouraging for the scale-up of CBP of FP in the remaining districts of Rwanda, which commenced in 2012. However, the fact that over two-thirds of intervention CHWs reported that one person in the binome pair is not sufficient to meet all of a village’s FP needs should be taken into account before wholesale implementation of the program. To maintain job satisfaction and keep the workload at a reasonable volume, the Ministry of Health may want to explore this issue further, not only with CHWs already providing CBP of FP, but also with those who have not yet been trained. In addition, it should be noted that CHWs in other countries are authorized to initiate clients on pills and injectables [14, 15] rather than just provide a resupply, as in Rwanda.

Although there was a difference in the services CHWs provided by intervention and control groups, both reported spending approximately the same amount of time performing their CHW duties. However, the greater amount of time the intervention group reported spending on meetings, training, and supervision may possibly be related to CBP supervision.

The discovery that cooperatives were not considered to be among the top three sources of motivations to work as a CHW was surprising. Income or livelihood-generating opportunities, along with non-financial incentives such as recognition by and having status in the community, are key considerations for motivating CHWs [13]. The Government of Rwanda presumed that the opportunity to generate income through this performance-based mechanism would figure more prominently as an incentive. Additional inquiries should be made to determine why cooperatives are not playing their intended role. They may need to be better supported to exact greater benefit for CHWs and their communities.

Ratings of satisfaction and service quality are often very high [16], as we found in client data. However, there was a noteworthy, albeit not significant, difference between client ratings for those indicators and the desire to receive services from CHWs in the future. Additional inquiries should be made to determine if the responses merely reflect the limitations of CHWs vis à vis clinic providers or if some aspects of CHW services could be improved.

The study was limited by the subjective nature of information recorded on CHW work-related activities in diaries. An observational study would have been optimal, but logistical, ethical and financial constraints precluded that methodology. Nevertheless, we believe that collecting the data from both control and intervention CHWs over a one-month period reduced potential noise. CHWs may have exaggerated the hours they worked or the clients they served in an effort to demonstrate their diligence, but there is no reason to believe that one group would have exaggerated more than the other. In addition, courtesy bias may have elevated reported satisfaction from both clients and CHWs, but again, there is no reason to suspect differences in reporting by intervention or control group.

This study set out to answer the practical question of whether or not the addition of pill and injectable re-supply would affect the quality of services provided by binomes in Rwanda. Results, based not only on the documented volume of work, but by CHW and client perceptions as well, strongly suggested that they would not adversely affect service quality or the relationships between CHWs and clients.
